# BPT-PLR: A Balanced Partitioning and Training Framework with Pseudo-Label Relaxed Contrastive Loss for Noisy Label Learning

**DOI:** 10.3390/e26070589

**Published:** 2024-07-10

**Authors:** Qian Zhang, Ge Jin, Yi Zhu, Hongjian Wei, Qiu Chen

**Affiliations:** 1 School of Information Technology, Jiangsu Open University, Nanjing 210036, China; zhangqian@jsou.edu.cn (Q.Z.); jinge@jsou.edu.cn (G.J.); zhuyi@jsou.edu.cn (Y.Z.); 2 School of Communication & Information Engineering, Shanghai University, Shanghai 200444, China; 3 School of Physics and Electronic Engineering, Fuyang Normal University, Fuyang 236037, China; weihongjian@fynu.edu.cn; 4 Department of Electrical Engineering and Electronics, Graduate School of Engineering, Kogakuin University, Tokyo 163-8677, Japan

**Keywords:** deep neural networks, noisy labels, pseudo-label relaxed contrastive loss, Gaussian mixture model

## Abstract

While collecting training data, even with the manual verification of experts from crowdsourcing platforms, eliminating incorrect annotations (noisy labels) completely is difficult and expensive. In dealing with datasets that contain noisy labels, over-parameterized deep neural networks (DNNs) tend to overfit, leading to poor generalization and classification performance. As a result, noisy label learning (NLL) has received significant attention in recent years. Existing research shows that although DNNs eventually fit all training data, they first prioritize fitting clean samples, then gradually overfit to noisy samples. Mainstream methods utilize this characteristic to divide training data but face two issues: class imbalance in the segmented data subsets and the optimization conflict between unsupervised contrastive representation learning and supervised learning. To address these issues, we propose a Balanced Partitioning and Training framework with Pseudo-Label Relaxed contrastive loss called BPT-PLR, which includes two crucial processes: a balanced partitioning process with a two-dimensional Gaussian mixture model (BP-GMM) and a semi-supervised oversampling training process with a pseudo-label relaxed contrastive loss (SSO-PLR). The former utilizes both semantic feature information and model prediction results to identify noisy labels, introducing a balancing strategy to maintain class balance in the divided subsets as much as possible. The latter adopts the latest pseudo-label relaxed contrastive loss to replace unsupervised contrastive loss, reducing optimization conflicts between semi-supervised and unsupervised contrastive losses to improve performance. We validate the effectiveness of BPT-PLR on four benchmark datasets in the NLL field: CIFAR-10/100, Animal-10N, and Clothing1M. Extensive experiments comparing with state-of-the-art methods demonstrate that BPT-PLR can achieve optimal or near-optimal performance.

## 1. Introduction

Large-scale, accurately labeled image data are one of the key prerequisites for the success of deep neural networks (DNNs) in numerous computer vision (CV) tasks, such as image captioning [1], image classification [2], segmentation [3,4], etc. However, collecting these large-scale, high-quality annotated datasets requires significant manpower and resources. The current data collection process mainly involves scraping data from search engines, forums, and other websites and then relying on the help of a large number of annotation experts on crowdsourcing platforms (Amazon Mechanical Turk, etc.) to cross-check and correct the tags. This process is time-consuming and becomes more challenging as the dataset size increases, leading to partially inaccurate annotations (noisy labels) even after verification. A wealth of research has shown that due to the over-parameterization of DNNs, they attempt to fit labels for all samples, including noisy labels, severely compromising the generalization performance of DNNs. Therefore, existing research focuses on collecting data without relying on manual annotation and assisting DNNs in learning from noisy datasets. This aims to prevent overfitting to noisy samples while maintaining performance levels close to those achieved when learning from clean datasets, known as noisy label learning (NLL) research.

Existing research indicates that although DNNs eventually fit all samples, they initially fit the predominant clean-label samples in the dataset and gradually overfit the noisy-labeled samples [2,5]. This memorization characteristic of DNNs results in clean samples having smaller losses in the early stage, while noisy samples exhibit larger losses, hence termed the small-loss criterion [5], widely employed in methods learning from noisy labels. Centered around the memorization characteristic of DNNs and the small-loss criterion, existing NLL methods can be categorized into three types: robust training loss, label correction, and sample selection. The first two methods will be introduced in the next section. Due to the superior performance of recent sample selection methods, it is crucial to conduct relevant research. Our method can also be categorized in this direction. Early sample selection techniques utilize the small-loss criterion, selecting samples with smaller cross-entropy losses during training as a subset of clean labels for supervised training. However, these methods perform inferiorly due to the inadequate utilization of training data compared with other types of methods. With further advancement in NLL research, some sample selection methods have begun utilizing various loss distribution estimation methods (i.e., GMM, the beta mixed model) to partition training data based on the small-loss criterion, retaining observed labels of samples with smaller losses (labeled samples) and discarding labels of samples with larger losses (unlabeled samples). Subsequently, semi-supervised learning (SSL) techniques and contrastive representation learning (CRL) techniques are introduced to deeply train the partitioned training subsets and improve performance. Existing sample selection methods based on SSL techniques mostly derive from DivideMix, differing significantly in data partitioning techniques and semi-supervised training strategies aimed at enhancing model robustness. Although these methods have achieved certain results, their performance still has room for further improvement due to issues such as class imbalance in the partitioned subsets and optimization conflicts between contrastive representation losses and supervised losses. Although PLReMix has addressed some of these issues, there are still some challenges remaining. Compared to the original DivideMix, PLReMix primarily introduces a dual-component GMM based on sample semantic and category information for data partitioning during the sample selection process. Subsequently, the robust training process integrates the new pseudo-label relaxed contrastive loss (PLR) with existing SSL techniques. According to our analysis, this method faces two main issues: (1) During actual training, it is challenging for the model to completely avoid the influence of noisy labels in the early stages, resulting in many clean samples being mislabeled as noisy, especially in high-noise scenarios where PLReMix tends to generate a large number of false positives as depicted in Figure 3 under 90% symmetric noise and 40% and 49% asymmetric noise scenarios. (2) In the SSL training process, the number of model iterations per epoch depends on the size of the current labeled set. In high-noise scenarios, however, the number of labeled samples is much smaller than the unlabeled ones (as shown in Figure 5), preventing the model from fully learning the data distribution and thereby limiting performance improvements.

To address the issue in existing sample selection methods, we propose a framework named BPT-PLR (Balanced Partitioning and Training framework with Pseudo-Label Relaxed contrastive loss). This framework follows the structural design of existing sample selection methods based on SSL techniques such as DivideMix [6], PLReMix [7], LongReMix [8], and C2MT [9], but introduces two key processes: a balanced partitioning process with a two-dimensional Gaussian mixture model (BP-GMM) and a semi-supervised oversampling training process with a pseudo-label relaxed contrastive loss (SSO-PLR). Similar to PLReMix, our method employs a dual-component GMM during the BP-GMM process to model both the semantic and class information of samples. However, as shown in Figure 3, the divided labeled set is not entirely reliable. Furthermore, to mitigate the impact of class imbalance on model performance, we adopt a class-level balanced selection strategy to ensure that the number of samples in each class of the filtered labeled subset is as close as possible. Additionally, while CRL can enable the model to learn intrinsic semantic information of data independent of noisy labels, aiding in selecting samples containing noisy labels, it conflicts with the supervised loss (e.g., CE) when cooperating with SSL techniques. Therefore, the SSO-PLR process combines PLR with SSL techniques, obtaining more reliable negative pairs by checking whether the top K indices of prediction probabilities between different samples have an empty intersection. This preserves resistance to noisy labels and avoids conflicts with supervised loss. As mentioned above, the number of labeled samples is much smaller than the unlabeled ones. Therefore, we introduce oversampling techniques to overcome the problem of existing sample selection methods failing to fully exploit feature information from unlabeled samples during the SSL process. We validate its effectiveness on four benchmark datasets in the NLL domain, and extensive experiments demonstrate that compared with state-of-the-art (SOTA) methods, BPT-PLR can achieve similar or better test performances. The source code is available at https://github.com/LanXiaoPang613/BPT-PLR (accessed on 5 July 2024). Our main contributions are as follows:

1. We propose an improved end-to-end training framework called BPT-PLR (Balanced Partitioning and Training framework with Pseudo-Label Relaxed contrastive loss) to address issues of noisy label learning (NLL) in DNNs, such as class imbalance in partitioned subsets and optimization conflicts between CRL losses and supervised losses. This framework enhances DNNs’ robustness to noisy labels and achieves superior performance.
2. We introduce a novel class-level balanced selection method based on a two-dimensional Gaussian mixture model (GMM). This method first models both the semantic and class information of the data using a two-dimensional GMM and then utilizes a class-level balanced selection strategy based on the distribution of samples to partition the data. This ensures that the labeled subset after partitioning maintains class balance, thereby alleviating the impact of the long-tail issue on model accuracy.
3. We incorporate the existing PLR loss into a semi-supervised learning (SSL) framework following previous work but further leverage it through oversampling techniques. This process enhances the model’s learning of semantic information from both labeled and unlabeled samples, thereby improving test performance.
4. We demonstrate the effectiveness of BPT-PLR through extensive experiments on several classic datasets in the NLL field. Additionally, we validate the robustness of the two key processes proposed through ablation experiments.

The structure of this paper is outlined as follows: In Section 2, we introduce some existing research relevant to the method proposed in this paper. Section 3 is dedicated to introducing our method, while Section 4 provides a detailed explanation of the experiments and comparisons. Finally, we conclude in Section 6.

## 2. Related Works

This section mainly introduces recent research in the fields of noisy label learning (NLL) and contrastive representation learning (CRL).

### 2.1. Recent Research on NLL

Robust training loss. Due to the widely used cross-entropy (CE) loss in classification tasks causing DNNs to be prone to overfitting noisy labels, leading to poor generalization performance, many studies deliberately design losses that are insensitive and underfitting to noisy labels to substitute for cross-entropy during training. Since Natarajan et al. [10] proved that if the loss function satisfies the symmetry condition, it is robust to label noise, many studies were conducted around it. For instance, Zhang et al. [11] have demonstrated that while the Mean Absolute Error (MAE) exhibits robustness to noisy labels under symmetry conditions, this robustness can increase training difficulty and decrease model performance. Therefore, they combined CE with MAE to propose Generalized Cross Entropy (GCE) loss, which possesses not only the advantage of CE’s rapid convergence but also the robustness of MAE to noisy labels. Similarly, inspired by [12], Oaraei et al. [13] proposed a convex surrogate of the unbiased 0–1 loss for content recommendation and multimedia search tasks, which typically encounter issues of class imbalance and missing labels [14]. Additionally, inspired by the symmetric Kullback–Leibler (KL) divergence, Wang et al. [15] introduced Symmetric Cross-Entropy (SCE) loss and theoretically demonstrated its robustness to noisy labels under certain conditions. Zhang et al. [16] proposed a novel loss function called Mixup, which interpolates between any two samples according to a beta distribution and then computes the CE loss for the interpolated sample. This method has been widely adopted in the field of NLL. Recently, Ye et al. [17] integrated activation loss functions with strategies like supplementary label learning to devise a normalized negative loss function [18], replacing the MAE loss used in active–passive loss. This approach enables the model to focus more on learning clean samples. Additionally, Jain et al. presented a propensity-scored loss for extreme multi-label learning, which is useful for addressing tagging tasks and has the potential to be expanded to the task of pseudo-label generation in NLL research. However, as these functions are designed to underfit noisy labels, they also underfit a portion of clean samples that are difficult to distinguish, resulting in poor performance.

Label correction. The label correction process primarily leverages the memorization characteristic of DNNs, where after a certain time of pre-training, model predictions are used to replace observed labels of samples to alleviate the impact of noisy labels on model performance. The joint optimization framework [19] directly utilizes model prediction to replace original labels, which cannot finely update each sample, leading to model performance fluctuations. Yi et al. [20] proposed the PENCIL framework to continuously correct labels based on the gradients generated when each sample participates in loss computation and backpropagation, thus alleviating the fluctuation. Building upon this, Zhang et al. [2] introduced Mixup [16] and balance terms to enhance the label correction capability further and [21] proposed a novel label correction framework for feature-dependent label noise. Additionally, Xu et al. [22] introduced contrastive prototypical loss to maximize the distance between the class cluster and the data point and assist in the label correction process. Similarly, Huang et al. [23] employed supervised contrastive learning techniques to guide the label correction process, achieving certain improvements. Wang et al. [24] proposed an end-to-end dynamic correction method for NLL, which utilizes the knowledge from past epochs to combat label noise. However, these methods exhibit performance fluctuations when faced with real-world datasets, thus casting doubt on their practical utility.

Sample selection. The early sample selection methods only select samples with smaller losses for training to mitigate the impact of noisy labels. For example, Co-teaching [25] employs two networks to alternately select small loss samples for training, while CJC-net [26] eliminates noisy labels through cross-training and learning rate oscillation strategies. As research progresses, DivideMix [6] and ELR [27] pioneers combine SSL techniques with sample selection methods to fully utilize the information carried by both clean and noisy samples, achieving significant progress. Subsequently, Karim et al. [28] introduced unsupervised CRL and Jensen–Shannon divergence (JSD) into semi-supervised sample selection methods to further boost performance. Zhang et al. [29] proposed a new sample selection and weighting method called Hyper-spherical Margin Weighting (HMW) and embedded it into [28]. Feng et al. [30] applied optimal transport theory to the sample selection process. Li et al. [31] adopted different dynamic thresholds for selecting clean, challenging, and noisy samples, combined with semi-supervised learning techniques to improve performance. Additionally, Zhang et al. [9] further improved DivideMix by introducing cross-to-merge training strategies and median balance strategies to enhance performance. Cordeiro et al. [8] decomposed the sample selection and robust training processes of DivideMix into two steps for targeted optimization, achieving certain progress. Sun et al. [32] simplified the sample selection problem into a clustering problem and introduced twin contrastive clustering to resolve it. Deng et al. [33] proposed SLRLNL to separate noisy labels from hard yet clean samples to improve model robustness.

### 2.2. Recent Research on CRL

CRL is a representative self-supervised learning technique that can learn feature representations independent of labels. During training, positive and negative examples from a batch of data need to be constructed to calculate the InfoNCE loss. SimCLR [34] uses two strong data augmentations for each sample as positives, while considering other samples as negatives to compute InfoNCE, thus requiring larger batch sizes. Meanwhile, MoCo [35] utilizes a momentum encoder and a queue to generate negatives for samples, reducing the batch size. Additionally, Khosla et al. [36] extended self-supervised CRL to a fully supervised setting by leveraging label information, where samples of the same class are treated as positives and samples of different classes are treated as negatives. Li et al. [37] calculated the moving average low-dimensional embeddings of each class to obtain category prototypes and utilized these prototypes to perform CRL. Due to the capability of CRL to enable models to learn semantic information independent of labels in the data, it holds great potential for application in the NLL field. However, the labels of samples in noisy datasets are unreliable, resulting in fewer applications of supervised CRL [38]. Instead, many NLL methods introduce unsupervised CRL techniques to enhance the robustness of models to noisy labels [39]. However, ref. [7] demonstrated conflicting optimization between the contrastive loss computed using unsupervised CRL and the supervised loss computed using model output values and observed labels. This conflict limits further improvement in model testing accuracy. Therefore, they define reliable negative pairs as those where the intersection of the top K indices of predicted probabilities for any sample and the top K indices of a given sample is empty and utilize these negative pairs to compute CRL, reducing the optimization conflict between contrastive loss and supervised loss. However, PLReMix [7] requires using different types of similar PLR losses for different types of datasets (for example, using Flat PLR for CIFAR [40] and using native PLR for Clothing1M [41]), and the performance varies significantly. Although our method adopts the proposed PLR loss, we successfully overcome these challenges by introducing two key processes.

## 3. Algorithm

Common DNNs for classification tasks typically consist of a feature extractor $f\left(\cdot ,\theta \right)$ and a classifier $h\left(\cdot ,\varphi \right)$, where θ and φ are the corresponding learnable parameters. The feature extractor generates high-dimensional features $z=f\left(x,\theta \right)$ for any input x, while the classifier produces model predictions $h\left(z,\varphi \right)$ based on z. Therefore, with the assumption of training a *k*-class classification network on a dataset $D={\left\{\left(x_{i},y_{i}\right)\right\}}_{i=1}^{N}$ containing *N* samples, $x_{i}\in R^{H\times W}$ represents the *i*-th training instance and $y_{i}=\left[k\right]=\left\{1,2,\ldots ,k\right\}$ is the corresponding ground-truth (GT) label. Most classification tasks are performed using the CE loss as shown in Equation (1), minimizing $L_{ce}$ to fit the DNN to all given labels.

(1) $$L_{ce}=-\frac{1}{N}\sum_{i=1}^{N}\log\left(p\left(y_{i}\left|x_{i}\right.\right)\right),$$

where $p\left(y_{i}\left|x_{i}\right.\right)$ is the $y_{i}$-th component of the prediction $p\left(x_{i}\right)=\mathrm{softmax}\left(h\left(f\left(x_{i},\theta \right),\varphi \right)\right)$ for the input $x_{i}$. However, when there are mislabeled samples in the dataset, i.e., $y_{i}\neq {\tilde{y}}_{i}$ (let $\tilde{D}={\left\{\left(x_{i},{\tilde{y}}_{i}\right)\right\}}_{i=1}^{N}$ and ${\tilde{y}}_{i}=\left[k\right]$ denote the noisily labeled dataset and noisy label), from the perspective of gradient contribution [29,30], it has been shown that samples with noisy labels carry greater weight compared with those with clean labels as convergence progresses, rendering this paradigm unreliable [27]. Therefore, NLL emerges.

**Figure 1:**
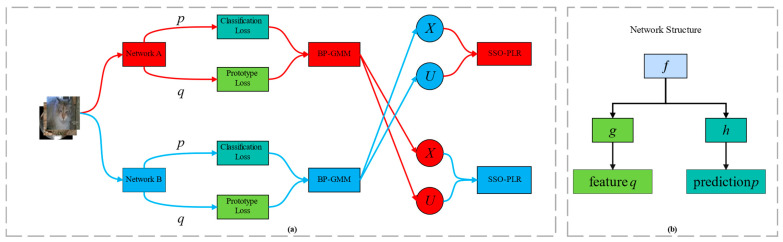
Overall framework of the BPT-PLR. (**a**) Overall process: The training data are fed into two networks, A and B, for loss computation. In each network, the extracted features are used to calculate prototype loss together with class prototypes, while the output predictions are used to compute classification loss and observed labels. Subsequently, the BP-GMM process (i.e., Section 3.1) utilizes the semantic and label information carried by these two losses to balance the partitioning of the training data. In this process, the labeled subset *X* and the unlabeled subset *U* partitioned by network A are used by network B for the SSO-PLR process (i.e., Section 3.2), and vice versa. (**b**) Network structure: Each network consists of a feature extractor *f* = *f*(⋅, *θ*^(*m*)^), a projection head *g* = *g*(⋅, *ϕ*^(*m*)^), and a classifier *h* = *h*(⋅, *φ*^(*m*)^), where *θ*^(*m*)^, *ϕ*^(*m*)^, and *φ*^(*m*)^ are the corresponding parameters, and *m* ∈ {0, 1} denotes the network index (e.g., *m* = 0 represents network A).

An overview of the proposed framework is shown in Figure 1. Our framework is similar to the existing sample selection with SSL techniques, employing two identical DNNs that are trained alternately. Like PLReMix [7], each DNN comprises a feature extractor $f\left(\cdot ,\theta^{\left(m\right)}\right)$ and a classifier $h\left(\cdot ,\varphi^{\left(m\right)}\right)$ for semi-supervised classification tasks, along with an additional projection head $g\left(\cdot ,\phi^{\left(m\right)}\right)$ to map high-dimensional features z to low-dimensional embedding q. Here, $\theta^{\left(m\right)}$, $\varphi^{\left(m\right)}$, and $\phi^{\left(m\right)}$ are the corresponding parameters, and $m\in \left\{0,\;1\right\}$ denotes the network index. We pre-train both models using CE loss. To address asymmetric noise scenarios, we introduce an additional penalty term [6,9,30,31] based on the prediction confidence to promote a more uniform loss distribution, facilitating GMM modeling. This penalty term for the *m*-th model is given as follows:

(2) $$L_{p}=-\frac{1}{N}\sum_{i=1}^{N}p^{\left(m\right)}\left(x_{i}\right)\cdot \log\left(p^{\left(m\right)}\left(x_{i}\right)\right).$$

Here, $p^{\left(m\right)}\left(x_{i}\right)=\mathrm{softmax}\left(h\left(f\left(x_{i},\theta^{\left(m\right)}\right),\varphi^{\left(m\right)}\right)\right)$ is the softmax prediction of the *m*-th network for the input $x_{i}$. In the next two sections, we will provide a detailed explanation of the two key processes discussed in this article, i.e., BP-GMM and SSO-PLR.

### 3.1. Balanced Partitioning Process

**Figure 2:**
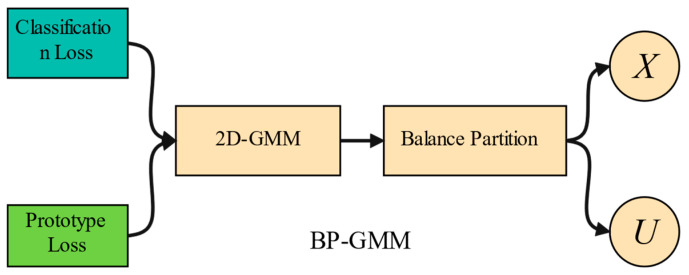
An overview of the BP-GMM process. Firstly, similar to PLReMix, a 2D GMM model is constructed based on the classification and prototype losses of all samples to estimate the posterior probability of each sample belonging to clean labels. Unlike PLReMix, to reduce the number of false positive samples in the labeled set and achieve a more balanced category distribution, class-level balanced selection is conducted based on the estimated probabilities to ensure the sample quantities of each class in the labeled subset *X* are close, ultimately resulting in the labeled subset *X* and the unlabeled subset *U*.

After the warm-up stage, at the beginning of each epoch, we first divide the entire dataset $\tilde{D}$ into a labeled set $X^{\left(m\right)}$ and an unlabeled set $U^{\left(m\right)}$ through this process for each network $m\in \left\{0,1\right\}$. In the labeled set $X^{\left(m\right)}$, the original label of each sample is considered to be nearly correct, so we retain its label; whereas, in the unlabeled set $U^{\left(m\right)}$, the original label of each sample is deemed incorrect, thus we remove its label to alleviate model overfitting. Then, we separately calculate the classification cross-entropy (CCE) loss and the prototype cross-entropy (PCE) loss for each sample under the two models to fit a two-component two-dimensional GMM. We use the GMM to estimate the posterior probability of samples being clean labels. Figure 2 illustrates the BP-GMM process.

Assuming we currently compute two types of losses based on model *m*, then the CCE is the de-mean of Equation (1) (e.g., $L_{ce,i}^{\left(m\right)}=-\log\left(p\left(y_{i}\left|x_{i}\right.\right)\right)$); the CCE measures how well the network fits sample labels, which is consistent with Equation (1), except that the GT label $y_{i}$ is replaced by the observed label ${\tilde{y}}_{i}$. Modeling the CCE of each sample using GMM can fully utilize the class information they carry. Furthermore, the PCE represents the semantic-level potential category probability distribution between the low-dimensional embedding $q_{i}^{\left(m\right)}$ of the sample $x_{i}$ and all class prototypes ${\left\{Q_{c}^{\left(m\right)}\right\}}_{c=1}^{k}$ under network *m*. Here, $Q_{c}^{\left(m\right)}$ is the prototype of the *c*-th class and is defined as the mean center of low-dimensional embeddings with the same semantic information. The initialization and update methods are detailed in Equations (18) and (21) of Section 3.3. Here, we assume that all class prototypes ${\left\{Q_{c}^{\left(m\right)}\right\}}_{c=1}^{k}$ for the current epoch have been obtained. Consequently, the PCE of instance $x_{i}$ is denoted as

(3) $$L_{pro,i}^{\left(m\right)}=-\sum_{c=1}^{k}{\tilde{y}}_{i}\log\left(d_{i}^{\left(m\right)}\right).$$

Here, ${\tilde{y}}_{i}$ is the one-hot representation of the observed label ${\tilde{y}}_{i}$ and $d_{i}^{\left(m\right)}={\left\{d_{i,c}^{\left(m\right)}\right\}}_{c=1}^{k}$ denotes the normalized cosine similarity matrix. Subsequently, the *c*-th component of $d_{i}^{\left(m\right)}$, can be calculated according to Equation (4):

(4) $$d_{i,c}^{\left(m\right)}=\frac{\exp\left(q_{i}^{\left(m\right)}\cdot Q_{c}^{\left(m\right)}/0.1\right)}{\sum_{j=1}^{k}\exp\left(q_{i}^{\left(m\right)}\cdot Q_{j}^{\left(m\right)}/0.1\right)}$$

Here, $d_{i,c}^{\left(m\right)}$ represents the distance between the embedding $q_{i}^{\left(m\right)}$ and $Q_{c}^{\left(m\right)}$, which is adopted from [7,37]. In an ideal scenario, under the influence of a proficient feature extractor $f\left(\cdot ,\theta^{\left(m\right)}\right)$ and projection head $g\left(\cdot ,\phi^{\left(m\right)}\right)$, the mapping embeddings of samples with similar semantic information should form a cluster, with the center of this cluster representing the corresponding class prototype. In such a case, if the given label ${\tilde{y}}_{i}$ for instance pair $\left(x_{i},{\tilde{y}}_{i}\right)\in \tilde{D}$ does not match its GT label $y_{i}$, then its distance $d_{i,{\tilde{y}}_{i}}^{\left(m\right)}$ corresponding to the observed label should be smaller than $d_{i,y_{i}}^{\left(m\right)}$. Consequently, the prototype cross-entropy loss for this instance would be greater than that for other instances with the same observed label where the observed label matches the true label. Therefore, the semantic information carried by training data can also be fully employed by fitting GMM to the PCE loss.

After obtaining these two types of losses based on model *m*, a two-component two-dimensional GMM is trained to fit the distribution $S^{\left(m\right)}={\left\{s_{i}^{\left(m\right)}\right\}}_{i=i}^{N}={\left\{\left(L_{ce,i}^{\left(m\right)},L_{pro,i}^{\left(m\right)}\right)\right\}}_{i=1}^{N}$. Since samples with clean labels typically have smaller losses, it has been confirmed in the literature [6,7,8,9] that the mean center of the loss distribution formed by them is closer to 0 compared to noisy samples. Therefore, following the small-loss criterion, after modeling the GMM, we choose the component with the smallest mean from the two components and utilize the corresponding Gaussian model to estimate the posterior probability of each sample having a clean label. Here, we denote the posterior probability of this pair $\left(x_{i},{\tilde{y}}_{i}\right)\in \tilde{D}$ as $w_{i}^{\left(m\right)}$. According to Equation (5), the posterior probabilities of samples for each class are sorted in descending order, and the sorted set of posterior probabilities at the class level is denoted as ${\left\{W_{c}^{\left(m\right)}\right\}}_{c=1}^{k}$,

(5) $$W_{c}^{\left(m\right)}=sort\left(\left\{w_{i}^{\left(m\right)}\left|{\tilde{y}}_{i}=c,\;\left(x_{i},y_{i}\right)\in \tilde{D}\right.\right\}\right).$$

Here, $sort\left(\cdot \right)$ is the sorting function in descending order, and only the samples with observed labels ${\left\{{\tilde{y}}_{i}\right\}}_{i=1}^{N}$ belonging to category *c* will be sorted into $W_{c}^{\left(m\right)}$. Subsequently, we determine whether the posterior probability of all samples exceeds the predefined threshold $\tau_{s}\in \left[0,1\right]$, and we count the number of samples exceeding $\tau_{s}$ as $N_{c}^{\left(m\right)}$:

(6) $$N_{c}^{\left(m\right)}=\sum_{i=1}^{N}1\left(w_{i}^{\left(m\right)}\geq \tau_{s}\right).$$

Here, $1\left(\cdot \right)$ is an indicator function that returns 1 only when the condition (e.g., $w_{i}^{\left(m\right)}\geq \tau_{s}$) is met. We perform sample selection at the class level, as shown in Equations (6) and (7), and only the top ${\left\{R_{c}^{\left(m\right)}\right\}}_{c=1}^{k}$ samples from $W_{c}^{\left(m\right)}$ are selected for the labeled set $X^{\left(m\right)}$:

(7) $$X^{\left(m\right)}={\left\{\left(x_{i},{\tilde{y}}_{i},w_{i}^{\left(m\right)}\right)|w_{i}^{\left(m\right)}\in W_{c}^{\left(m\right)}\left[0:R_{c}^{\left(m\right)}\right],\;\forall \left(x_{i},{\tilde{y}}_{i}\right)\in \tilde{D}\;and\;{\tilde{y}}_{i}=c\right\}}_{c=1}^{k}.$$

Here, $R_{c}^{\left(m\right)}$ represents the selected labeled samples within the *c*-th class and can be denoted as follows:

(8) $$R_{c}^{\left(m\right)}=\left\{\begin{array}{c} \begin{array}{cc} \frac{N_{c}^{\left(m\right)}}{k}, & if\;\sum_{i=1}^{N}1\left({\tilde{y}}_{i}=c\right)\leq \frac{N_{c}^{\left(m\right)}}{k} \end{array}\\ \begin{array}{cc} \sum_{i=1}^{N}1\left({\tilde{y}}_{i}=c\right), & otherwise \end{array} \end{array}\right..$$

The unlabeled set $U^{\left(m\right)}$ is obtained as follows:

(9) $$U^{\left(m\right)}={\left\{\left(x_{i},w_{i}^{\left(m\right)}\right)|w_{i}^{\left(m\right)}\in W_{c}^{\left(m\right)}\left[0:R_{c}^{\left(m\right)}\right],\;\forall \left(x_{i},{\tilde{y}}_{i}\right)\in \tilde{D}\backslash X^{\left(m\right)}\right\}}_{c=1}^{k}.$$

**Figure 3:**
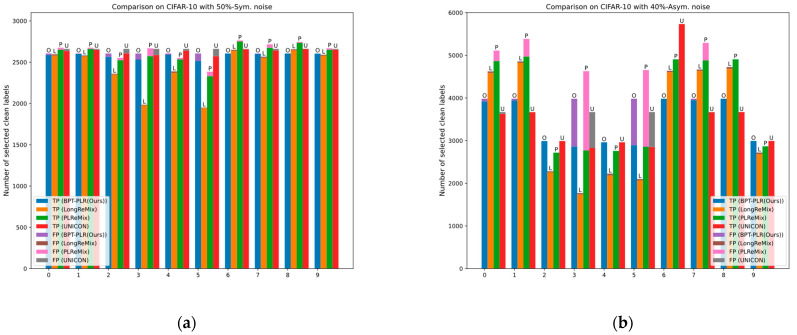

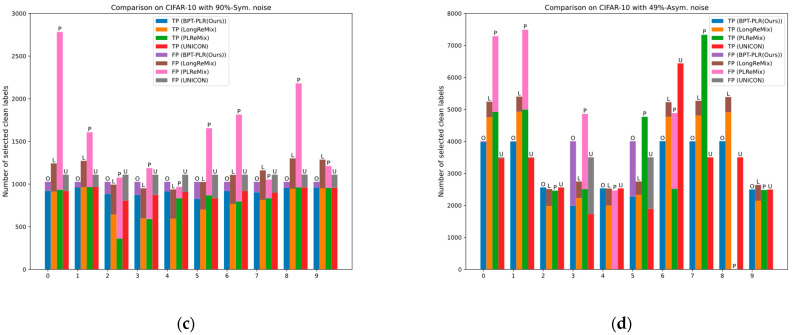
Efficiency comparison of sample selection methods using the CIFAR10 dataset at 100 epochs with different proportions of noisy labels. (**a**) Comparison using CIFAR10 dataset with 50% symmetric noisy labels. TP refers to clean samples correctly selected into the labeled set, while FP refers to noisy samples mistakenly included in the labeled set. L, P, U, and O represent LongReMix [8], PLReMix [7], UNICON [28] and our method, respectively. (**b**) Comparison using CIFAR10 dataset with 40% asymmetric noisy labels. (**c**) Comparison using CIFAR10 dataset with 90% symmetric noisy labels. (**d**) Comparison using CIFAR10 dataset with 49% symmetric noisy labels.

Previous methods [6,7,8,9] that used 1d-GMM or 2d-GMM to estimate posterior probabilities for sample partitioning have overlooked the class imbalance in the labeled subsets after the selection process. We propose a method called BP-GMM, which combines a balancing partition mechanism with a 2d-GMM to address this issue. As shown in Figure 3, we present the number of true positive (TP) and false positive (FP) samples within each class of the labeled subsets partitioned using BP-GMM and several representative methods (such as PLReMix [7], UNICON [28], and LongReMix [8]). From Figure 3, it is evident that BP-GMM not only maintains class balance in the partitioned labeled subsets but also increases the number of true positive samples in each class. Although UNICON also addresses class imbalance, resulting in balanced samples after selection, its use of Jensen–Shannon divergence (JSD) to partition based solely on class information neglects semantic information. Even so, in the selected labeled subset, the number of TP samples is significantly lower compared with the results obtained using our method, except for the 50%-sym. scenario, where the results of the two methods are close.

After partitioning the labeled subsets and the unlabeled subsets based on two models using the BP-GMM process, as illustrated in Figure 1a, the two subsets divided by the *m*-th model will be utilized in the SSL training of the (1-*m*)-th model in the SSO-PLR process. Similarly, the *m*-th model employs the two subsets divided by the (1-*m*)-th model for SSL training. Through this co-teaching strategy, the accumulation of error flows for each model is significantly alleviated [6,25,26]. The following section will explain the SSO-PLR process and the initialization and updating methods of class prototypes.

### 3.2. Semi-Supervised Oversampling Training Process

**Figure 4:**
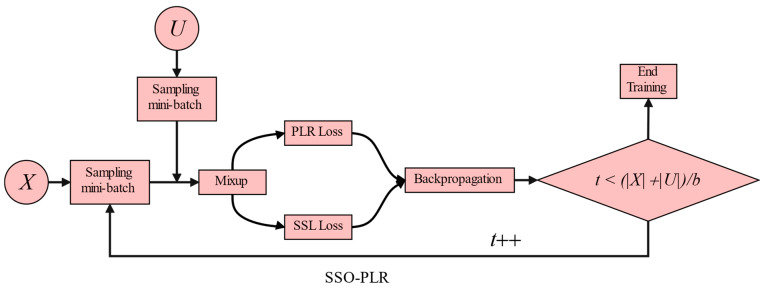
An overview of the SSO-PLR process. First, we sample a mini-batch of size b from the labeled dataset X and set the sampling count t to 1. Then, we sample a mini-batch of the same size from the unlabeled dataset U. Pseudo-labels are generated for both batches, followed by a Mixup operation to enhance the model’s generalization performance. Next, we compute the PLR loss and SSL loss and perform backpropagation. In this process, different from PLReMix, we introduce an oversampling mechanism to fully exploit feature information from unlabeled samples during the SSL process. If the sampling count t for the labeled dataset has not reached the maximum sampling times of the original dataset $\left(\left|X\right|+\left|U\right|\right)/b$, even if we have completed sampling the entire labeled dataset, we resample the labeled subset and increment t, continuing training to learn the remaining samples in the unlabeled subset. Training stops for the current epoch *e* only when $t\geq \left(\left|X\right|+\left|U\right|\right)/b$.

In this section, we illustrate the details of the SSO-PLR process. As shown in Figure 1a, we alternately train two models. Assuming the current training is for the *m*-th network, the two subsets, $X^{\left(1-m\right)}$ and $U^{\left(1-m\right)}$, that it uses are derived from the partition results of the (1−*m*)-th network. As illustrated in Figure 4, we employ an SSL framework similar to the previous sample selection methods [6,7,8,9] but with the addition of an oversampling strategy and PLR loss to further enhance the robustness and classification performance of the networks.

SSL loss. Taking the labeled subset $X^{\left(1-m\right)}$ as the primary sampling target, we first sample a mini-batch $B_{l}^{t}={\left\{\left(x_{i},{\tilde{y}}_{i},w_{i}^{\left(1-m\right)}\right)\right\}}_{i=1}^{b}$ of size *b* from it and a mini-batch $B_{ul}^{t}={\left\{\left(x_{i},w_{i}^{\left(1-m\right)}\right)\right\}}_{i=1}^{b}$ of the same size from the unlabeled subset $U^{\left(1-m\right)}$. Here, t represents the count of batch sampling from the labeled set $X^{\left(1-m\right)}$ in the current epoch e. As depicted in Figure 2, although the majority of samples in the labeled subset are clean, it unavoidably introduces some instances with noisy labels, leading to incomplete label reliability. Additionally, the original labels of samples in the unlabeled subset are unreliable. Hence, after applying weak augmentation $wk\left(\cdot \right)$ twice to each input $x_{i}$ in the two mini-batches, we generate pseudo-labels ${\hat{y}}_{i}$ for them using Equation (10).

(10) $$\left\{\begin{array}{c} {\hat{y}}_{i}=sp\left(w_{i}^{\left(1-m\right)}\cdot {\tilde{y}}_{i}+w_{i}^{\left(1-m\right)}\cdot \left(p^{\left(m\right)}\left(wk\left(x_{i}\right)\right)+p^{\left(m\right)}\left(wk\left(x_{i}\right)\right)\right)\right),\;\forall \left(x_{i},{\tilde{y}}_{i},w_{i}^{\left(1-m\right)}\right)\in B_{l}^{t}\\ {\hat{y}}_{i}=sp\left(\sum_{h=0}^{1}\left(p^{\left(h\right)}\left(wk\left(x_{i}\right)\right)+p^{\left(h\right)}\left(wk\left(x_{i}\right)\right)\right)\right),\;\forall \left(x_{i},w_{i}^{\left(1-m\right)}\right)\in B_{ul}^{t} \end{array}\right.$$

Here, ${\tilde{y}}_{i}$ is the one-hot representation of ${\tilde{y}}_{i}$ and $sp\left(\cdot \right)$ is a sharpen function used in previous works. The calculation of this sharpen function is as follows:

(11) $$sp\left({\bar{y}}_{i}\right)=\frac{{\left({\bar{y}}_{i}\right)}^{T}}{\sum_{j=1}^{k}{\left({\bar{y}}_{ij}\right)}^{T}},$$

where ${\bar{y}}_{ij}$ is the *j*-th component of the soft label ${\bar{y}}_{i}$ and *T* is the sharpening coefficient that is preset to 0.5. Next, we apply two rounds of strong augmentation (i.e., $stg\left(\cdot \right)$) to each input $x_{i}$ from $B_{l}^{t}$ and $B_{ul}^{t}$, respectively, and concatenate the augmented results in sequence to form two new batches $B_{l,stg}^{t}=\left\{\left(x_{i}^{stg},{\hat{y}}_{i}\right)\left|x_{i}^{stg}=stg\left(x_{i}\right),\;\forall \left(x_{i}^{stg},{\hat{y}}_{i}\right)\in B_{l}^{t}\right.\right\}$ and $B_{ul,stg}^{t}=\left\{\left(x_{i}^{stg},{\hat{y}}_{i}\right)\left|x_{i}^{stg}=stg\left(x_{i}\right),\;\forall \left(x_{i}^{stg},{\hat{y}}_{i}\right)\in B_{ul}^{t}\right.\right\}$. We then apply the Mixup [16] operation to each pair from the union of $B_{l,stg}^{t}$ to improve the models’ generalization and robustness, which is shown as follows:

(12) $$\left(x_{i}^{\prime },{\hat{y}}_{i}^{\prime }\right)=\lambda \cdot \left(x_{i}^{stg},{\hat{y}}_{i}\right)+\left(1-\lambda \right)\cdot \left(x_{i}^{stg},{\hat{y}}_{j}\right).$$

Here, the Mixup operation results for the input pair $\left(x_{i}^{stg},{\hat{y}}_{i}\right)$ are denoted as $\left(x_{i}^{\prime },{\hat{y}}_{i}^{\prime }\right)$, where λ is a dynamic value randomly sampled from the beta distribution $\mathrm{Beta}\left(\beta \right)$ with a predefined factor β and *j* represents a random permutation of the indices in $B_{l,stg}^{t}$ and $B_{ul,stg}^{t}$. Hence, we can denote the results in Equation (12) from two batches $B_{l,stg}^{t}$ and $B_{ul,stg}^{t}$ as $B_{l,mix}^{t}=\left\{\left(x_{i}^{\prime },{\hat{y}}_{i}^{\prime }\right)\left|x_{i}^{stg}\in B_{l,stg}^{t}\right.\right\}$ and $B_{ul,mix}^{t}=\left\{\left(x_{i}^{\prime },{\hat{y}}_{i}^{\prime }\right)\left|x_{i}^{stg}\in B_{ul,stg}^{t}\right.\right\}$, respectively. Subsequently, we compute the semi-supervised loss for each pair from $B_{l,mix}^{t}$ and $B_{ul,mix}^{t}$ as follows:

(13) $$\begin{array}{l} L_{ssl}^{\left(m\right)}=\underset{\mathrm{supervised}\;\mathrm{loss}}{\underset{︸}{-\frac{1}{\left|B_{l,mix}^{t}\right|}\cdot \sum_{\left(x_{i}^{\prime },{\hat{y}}_{i}^{\prime }\right)\in B_{l,mix}^{t}}\left({\hat{y}}_{i}^{\prime }\cdot \log\left(p^{\left(m\right)}\left(x_{i}^{\prime }\right)\right)\right)}}+\underset{\mathrm{unsupervised}\;\mathrm{loss}}{\underset{︸}{\frac{\lambda_{u}}{\left|B_{ul,mix}^{t}\right|}\cdot \sum_{\left(x_{i}^{\prime },{\hat{y}}_{i}^{\prime }\right)\in B_{ul,mix}^{t}}{\left\|{\hat{y}}_{i}^{\prime }-p^{\left(m\right)}\left(x_{i}^{\prime }\right)\right\|}_{2}^{2}}}\\ \underset{\mathrm{regularzation}\;\mathrm{loss}}{\underset{︸}{-\frac{1}{k}\cdot \log\left(\frac{1}{\left|B_{l,mix}^{t}\cup B_{ul,mix}^{t}\right|}\sum_{x_{i}^{\prime }\in B_{l,mix}^{t}\cup B_{ul,mix}^{t}}p^{\left(m\right)}\left(x_{i}^{\prime }\right)\right)}} \end{array}.$$

PLR loss. Currently, some sample selection methods not only employ Equation (13) to train networks but also utilize additional unsupervised CRL techniques to learn each pair from $B_{ul}^{t}$. In unsupervised CRL, each sample’s two transformations are treated as positive pairs, while transformations of all other samples in the batch serve as negative pairs. By leveraging InfoNCE [34,35], the similarity of positive embeddings is enhanced, while that of negative embeddings is diminished. However, in the batch, there might be some negative embeddings that align with the GT labels of the positive embeddings. In such cases, unsupervised CRL attempts to increase the distance between these negative embeddings and the positive embeddings, leading to a conflict with the optimization goal in Equation (13). Ref. [7] has demonstrated that this conflict significantly affects network performance. Therefore, this paper introduces PLR loss to help the feature extractor better learn from unlabeled information without disturbing the classifier. For the input $x_{i}^{\prime }$, following the method described in reference [7], we construct a reliable negative sample set $O_{i}^{t}$ (illustrated in Equation (14)) by removing instances from the union of $B_{l,mix}^{t}$ and $B_{ul,mix}^{t}$ that have the same potential GT labels as $x_{i}^{\prime }$. Given the model’s prediction $p^{\left(m\right)}\left(x_{i}\right)$ (without augmentation and Mixup operation) for input $x_{i}^{\prime }$ (it is the result of Mixup operation for $x_{i}$, the same index as $x_{i}$ in the dataset $\tilde{D}$), we first determine the top *n* indices with the highest prediction probabilities of $p^{\left(m\right)}\left(x_{i}\right)$ and their corresponding observed labels ${\tilde{y}}_{i}$, denoted as $top_{n}^{i}=\underset{n}{\arg\max}{\left\{p^{\left(m\right)}\left(c\left|x_{i}\right.\right)\right\}}_{c=1}^{k}\cup {\tilde{y}}_{i}$. Then, we include all instances from the union of $B_{l,mix}^{t}$ and $B_{ul,mix}^{t}$ in $O_{i}^{t}$ according to Equation (14).

(14) $$O_{i}^{t}=\left\{j\left|top_{n}^{i}\cap top_{n}^{j}=Ø,\forall j\in \left\{1,2,\ldots ,\left|B_{l,mix}^{t}\right|+\left|B_{ul,mix}^{t}\right|\right\}\backslash i\right.\right\}.$$

Subsequently, we compute the PLR loss using vanilla InfoNCE based on this negative sample set:

(15) $$L_{plr}=-\sum_{x_{i}^{\prime }\in B_{l,mix}^{t}\cup B_{ul,mix}^{t}}\frac{\exp\left(\langle q_{i}^{\left(m\right)},q_{+}^{\left(m\right)}\rangle /\tau_{p}\right)}{\exp\left(\langle q_{i}^{\left(m\right)},q_{+}^{\left(m\right)}\rangle /\tau_{p}\right)+\sum_{j\in O_{i}^{t}}\exp\left(\langle q_{i}^{\left(m\right)},q_{j}^{\left(m\right)}\rangle /\tau_{p}\right)}.$$

The final optimization objective of SSO-PLR is:

(16) $$L=L_{ssl}+L_{plr}.$$

**Figure 5:**
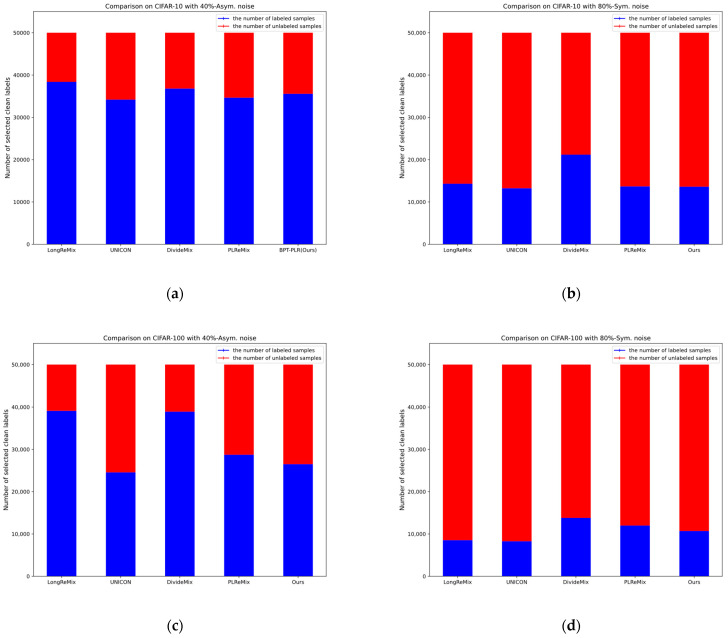
Comparison of labeled and unlabeled sample quantities partitioned by various sample selection methods. (**a**) Comparison using CIFAR-10 with 40% asymmetric noise labels. (**b**) Comparison using CIFAR-10 with 80% symmetric noise labels. (**c**) Comparison using CIFAR-100 with 40% asymmetric noise labels. (**d**) Comparison using CIFAR-100 with 50% symmetric noise labels. It is evident that as the noise rate increases (e.g., from CIFAR-10 with 40%-asym. To 80% sym., and CIFAR-100 with 40%-asym. To 50%-sym., etc.), the quantity of unlabeled samples significantly surpasses that of labeled samples. It must be noted that this tendency becomes more pronounced as the number of categories Increases (e.g., from CIFAR-10 with 80%-sym. To CIFAR-100 with 50%-sym.).

Oversampling. Existing semi-supervised sample selection methods sample a labeled mini-batch from $X^{\left(1-m\right)}$ with a predefined batch size *b* during training, then sample an unlabeled mini-batch from $U^{\left(1-m\right)}$ with the same size. After the Mixup operation, CRL loss (if any) and SSL loss are computed and backpropagated. Training of the current epoch e ends immediately when all labeled samples have been sampled, i.e., $t≜\left|X^{\left(1-m\right)}\right|/b$. However, in most noisy scenarios, due to the much larger size of the unlabeled subset partitioned $U^{\left(1-m\right)}$ by sample selection methods compared with the labeled subset $X^{\left(1-m\right)}$ (as shown in Figure 5), training is interrupted before many unlabeled samples are sampled, causing the model to miss the opportunity to learn from a large amount of unlabeled sample information [8].

Consequently, as shown in Figure 4, we introduce an oversampling mechanism. If the current sampling count *t* has not reached the maximum sampling times of the original dataset $\left(\left|X\right|+\left|U\right|\right)/b$, we continue sampling from the labeled and unlabeled sets, and training for the current epoch only stops when all unlabeled samples have been trained at least once. Resampling the clean data subset not only allows the model to learn information carried by all unlabeled samples but also reduces the boundary for vicinal risk minimization (as shown in Theorem 8 of [42], Section 5.4 of [8]), thereby enhancing the accuracy and robustness of semi-supervised classification.

### 3.3. Calculation of Class Prototypes

We maintain a class prototype set ${\left\{Q_{c}^{\left(m\right)}\right\}}_{c=1}^{k}$ for each model $m=\left\{0,1\right\}$. Let us assume the current model is the *m*-th one. After a certain number of training iterations (usually more than 10 epochs), this network has preliminarily converged and demonstrates basic classification performance. As class prototypes are defined as the mean centers of low-dimensional embeddings with the same semantic information, these embeddings form clusters around their corresponding class prototypes. Following prior research [7,37], at the end of the warm-up, we partition all samples from the whole noisy dataset $\tilde{D}$ into *k* subsets ${\left\{{\tilde{D}}_{c}\right\}}_{c=1}^{k}$ based on their observed labels ${\left\{{\tilde{y}}_{i}\right\}}_{i=1}^{N}$, which is shown in Equation (17):

(17) $${\tilde{D}}_{c}=\left\{\left(x_{i},{\tilde{y}}_{i}\right)\left|{\tilde{y}}_{i}=c,\;\forall \left(x_{i},{\tilde{y}}_{i}\right)\in \tilde{D}\right.\right\}$$

Then, for each subset ${\tilde{D}}_{c}$, the low-dimensional embeddings extracted by the feature extractor $f\left(\cdot ,\theta^{\left(m\right)}\right)$ and the projection head $g\left(\cdot ,\phi^{\left(m\right)}\right)$ are accumulated and averaged to form the corresponding class prototype $Q_{c}^{\left(m\right)}$, as expressed below:

(18) $$Q_{c}^{\left(m\right)}=\sum_{x_{i}\in {\tilde{D}}_{c}}g\left(f\left(x_{i},\theta^{\left(m\right)}\right),\phi^{\left(m\right)}\right).$$

At the end of each epoch after warm-up, we update all class prototypes ${\left\{Q_{c}^{\left(m\right)}\right\}}_{c=1}^{k}$ using the momentum updating method. First, we utilize the predictions $p^{\left(m\right)}\left(x_{i}\right)$ of the model *m* and the similarity $d_{i}^{\left(m\right)}$ measured by Equation (4) for estimating the latent GT labels:

(19) $$\delta_{i}=\alpha \cdot p^{\left(m\right)}\left(x_{i}\right)+\left(1-\alpha \right)\cdot d_{i}^{\left(m\right)},$$

where $\delta_{i}$ is the estimated latent label for the input $x_{i}$ and α=0.5 is a predefined coefficient to control the contribution of predictions for estimated labels. This process aims to maximize the utilization of information from both label and feature spaces. Subsequently, we utilize Equation (20) to determine the true classes of samples and select high-confidence samples to update class prototypes, aiming to further mitigate the impact of noisy samples on class prototype updates.

(20) $$y_{i}^{pro}=\left\{\begin{array}{l} \arg\;\max\;\delta_{i},\;\;\;\mathrm{if}\max\left(\delta_{i}\right)>\upsilon \\ \arg\;\max\;p^{\left(m\right)}\left(x_{i}\right),\;\mathrm{otherwise}\; \end{array}\right..$$

Here, υ=0.8 is a fixed threshold for performing label correction. Consequently, we update the class prototypes $Q_{y_{i}^{pro}}^{\left(m\right)}$ using the embedding $q_{i}^{\left(m\right)}$ and the estimated hard label $y_{i}^{pro}$, which is shown as follows:

(21) $$Q_{c}^{\left(m\right)}\leftarrow \mathrm{Norm}\left(\varsigma \cdot Q_{c}^{\left(m\right)}+\left(1-\varsigma \right)\cdot \mathrm{mean}\left(\sum_{x_{i}\in \tilde{D}}1\left(y_{i}^{pro}=c\right)\cdot q_{i}^{\left(m\right)}\right)\right).$$

Here, ς=0.99 is the momentum coefficient, $\mathrm{Norm}\left(\cdot \right)$ represents the normalization function, and $\mathrm{mean}\left(\cdot \right)$ is the mean function.

It should be noted that momentum updates are not performed during the warm-up stage (only initializing the class prototypes for each network according to Equation (18) after warm-up). After the SSO-PLR training process of each network, we sequentially execute the momentum update process for class prototypes as described in Equation (21).

### 3.4. Pseudo-Code

The pseudo-code of our method is illustrated in Algorithm 1, and an overall framework is shown in Figure 1.
**Algorithm 1:** Training process pseudo-code representation**Input:** $\mathrm{two}\;\mathrm{networks}\;m=0\;\mathrm{and}\;m=1;\;\mathrm{the}\;\text{warm-up}\;\mathrm{epochs}\;E_{w}$$;\;\mathrm{the}\;\mathrm{total}\;\mathrm{training}\;\mathrm{epochs}\;E_{tot}$$;\;\mathrm{batch}\;\mathrm{size}\;b;\;\mathrm{learning}\;\mathrm{rate}\;\mathrm{lr};\;\mathrm{thresholds}\;\tau_{s}$ and υ; epoch counter *e* = 0; sampling counter *t* = 0;
**while** $e<E_{tot}$ **do:**
  **if**
$e<E_{w}$**:**
    //enable Equation (2) only in the presence pf asymmetric noise labels
    pretrain the two networks on the whole dataset $\tilde{D}$ using Equations (1) and (2);
    **if** $e=\left(E_{w}-1\right)$:
      $\mathrm{initialize}\;\mathrm{class}\;\mathrm{prototypes}\;{\left\{Q_{c}^{\left(m\right)}\right\}}_{c=1}^{k}$ for each network using Equation (18); //It is the same as PLReMix
    **end if**
  **else:**
    re-initialize the sampling counter *t* = 0;
    //execute the BP-GMM process using from Equation (3) to Equation (9)
    //for network *m* = 0
    perform coarse data division using two-dimensional GMM (Equation (3) to Equation (4)); //It is the same as PLReMix
    perform the proposed class-level balanced selection on the coarse division results using (Equations (5)–(9)); //It is different from PLReMix
    $\mathrm{generate}\;\mathrm{labeled}\;\mathrm{subset}\;X^{\left(0\right)}$$\;\mathrm{and}\;\mathrm{unlabeled}\;\mathrm{subset}\;U^{\left(0\right)}$;
    //for network *m* = 1
    perform coarse data division using two-dimensional GMM (Equations (3) and (4)); //It is the same as PLReMix
    perform the proposed class-level balanced selection on the coarse division results using (Equations (5)–(9)); //It is different from PLReMix
    $\mathrm{generate}\;\mathrm{labeled}\;\mathrm{subset}\;X^{\left(1\right)}$$\;\mathrm{and}\;\mathrm{unlabeled}\;\mathrm{subset}\;U^{\left(1\right)}$;
    //execute the SSO-PLR process
    **for** network *m* = 0 **to** 1**:**
      **if**
$t<\left(\left|X^{\left(1-m\right)}\right|+\left|U^{\left(1-m\right)}\right|\right)/b$:      //oversampling strategy, it is different from PLReMix

      $\mathrm{sampling}\;a\;\mathrm{labeled}\;\text{mini-batch}\;B_{l}^{t}$
$\;\mathrm{and}\;\mathrm{an}\;\mathrm{unlabeled}\;\text{mini-batch}\;B_{l}^{t}$$\;\mathrm{from}\;X^{\left(1-m\right)}$$\;\mathrm{and}\;U^{\left(1-m\right)}$, respectively;
      perform label-refinement and co-guessing operation using Equation (10); //generate pseudo labels for all samples
      do Mixup augmentation for two mini-batches using Equation (12);    //enhance model generalization and robustness
      calculate the SSL loss and PLR loss through Equations (13) and (15);
      perform backpropagation according to Formula (14) to update all parameters of current network;
      t++;    //the increment of *t*
    **end if**    //all the unlabeled samples are completely sampled
  **end for**
  //update all the class prototypes, it is the same as PLReMix
  **for** network *m* = 0 **to** 1**:**

    estimate latent GT labels based on current network using Equations (19) and (20);
    perform momentum updates for the class prototypes belonging to the current network using Equation (21);
  **end for**
  e++;    //the increment of epoch counter *e*
**end while**
**Output:**
$\;\mathrm{two}\;\mathrm{robust}\;\mathrm{networks}\;m=0,\;1;\;\mathrm{two}\;\mathrm{labeled}\;\mathrm{subsets}\;X^{\left(m\right)}$ with relatively low noise rates.

## 4. Experiments

### 4.1. Datasets and Experimental Settings

Following previous research, such as [6,7,8,9,30,31], etc., we validated the performance of our approach on two synthetic noisy datasets (i.e., CIFAR-10 and CIFAR-100) and two real-world noisy datasets (i.e., Animal-10N and Clothing1M). The experiments covered various noise scenarios, and both coarse-grained and fine-grained datasets were validated. For the backbone (i.e., feature extractor f and classifier h) used in each dataset, we introduce an additional projection head g comprising two linear layers and one normalization layer. This head aims to transform the features outputted by the penultimate layer of the backbone network into a low-dimensional space of dimension 128, aiming to obtain a more compact embedding. The summary of the datasets used in this paper is demonstrated as follows:

CIFAR-10 [40]. The basic information of this dataset is shown in Table 1. Since all labels in the dataset are accurate (clean), we consider two types of synthetic noise labels: symmetric and asymmetric. By artificially synthesizing noisy labels, we can simulate scenarios such as label errors or confusion in the real world, thereby evaluating and improving the robustness of NLL methods in noisy environments. Symmetric noise randomly flips the labels of r% (i.e., noise rate) samples from each class to all other classes in a uniform distribution. Asymmetric noise simulates label confusion scenarios, mainly by flipping r% truck class samples to automobile, r% bird class samples to airplane, interchanging samples between the cat and dog categories, etc. We considered five symmetric noise scenarios, where r% takes values of 20%, 50%, 80%, and 90%, as well as four asymmetric noise scenarios, where r% takes values of 10%, 20%, 30%, 40%, and 49%. To ensure a fair comparison with previous methods, we employed the PreAct ResNet-18 [43] as the backbone. Table 2 shows the experimental settings of the method in this paper. To illustrate the robustness of our approach, we employed nearly identical parameter configurations across all noise scenarios. Despite prior studies suggesting that the parameter $\lambda_{u}$ should vary depending on noise rates and types, we opted for a fixed value of $\lambda_{u}=30$. The only exception occurred in low noise rate scenarios, such as 20%-sym. And 10% to 30%-asym., etc., where we set $\lambda_{u}=0$. This approach aligns with common sense, as lower noise rates should correspond to weaker regularization capabilities for unlabeled samples. Additionally, the learning rate *lr* linearly decays to 2 × 10^−4^ within the first 380 epochs and remains fixed thereafter. We decrease the *n* used in $top_{n}^{i}$ from 3 to 2 after 40 epochs.

CIFAR-100 [40]. The basic information of this dataset is also shown in Table 1. Following previous studies, we still consider both symmetric and asymmetric noise labels in this dataset. The generation of symmetric noisy labels is consistent with CIFAR-10 while the generation of asymmetric noisy labels involves flipping r% of samples from each category to the next similar category within its superclass. We considered r= 20, 50, 80, and 90 for symmetric noise scenarios and 10, 20, 30, and 40 for asymmetric scenarios. Table 2 shows the experimental settings in this paper. The $\lambda_{u}$ is still fixed as 30, except for 10% -asym. And 90%-sym., where $\lambda_{u}=0$ and 150, respectively. The adjustment of the learning rate *lr* is the same as CIFAR-10, comprehensively demonstrating the robustness of our method. The setting of *n* is the same as CIFAR-10.

Animal-10N [44]. This is a fine-grained real-world noise dataset, comprising 10 classes of animal data. The noise rate of this dataset is approximately 8%. The basic information is outlined in Table 1. The Vgg-19N [45] is utilized as the backbone. Table 2 shows the experimental settings in this paper. To illustrate the robustness of our approach, we employed nearly identical parameters to CIFAR-10 and set $\lambda_{u}$ to 0. The learning rate *lr* was reduced by 10 and 100 after 80 and 140 epochs, respectively. The setting of *n* was the same as CIFAR-10. Additionally, to ensure a fair comparison with some co-teaching-based methods, we also present experimental results based on the 9-layer CNN [25,26]. The hyperparameter settings of this backbone are identical to those of VGG-19N, demonstrating that our approach is insensitive to model architecture.

Clothing1M [41]. This is also a real-world noise dataset with nearly 38.4% noisy labels, comprising 14 categories of clothing images. Table 1 illustrates the summary of this set. ResNet-50 [43] pretrained with the ImageNet dataset is the backbone. Table 2 shows the experimental settings in this paper. Due to our adherence to previous methods that randomly balanced sampled 64K data for training in each epoch, during training, we added PLR loss to pre-train the projection head and feature extractor. Additionally, we performed model performance calibration using CE loss every 5 epochs. The hyperparameters were the same as Animal-10N. The learning rate *lr* was reduced by 10 per 40 epochs. We decreased the *n* from 3 to 2 and 1 after 15 and 30 epochs, respectively, which is also the same as [7].

### 4.2. Experiments on Synthetic Noisy Datasets

This section illustrates the performance variations in BPT-PLR on the CIFAR-10 and CIFAR-100 across various noise types and rates and compares our method with various SOTA methods from 2018 to 2024. All the results of our method are the means of two independent experiments.

#### 4.2.1. Results on CIFAR-10

Following the validation methodology established in the NLL field, we demonstrate the robustness and generalization of our method on CIFAR-10 using synthetic symmetric and asymmetric noisy labels. Table 3 presents the comparison of our method and some SOTA methods on CIFAR-10 with various noise types and rates. To demonstrate our approach’s robustness, we provide the average test accuracy of the last 10 epochs (denoted as the last) and the best test accuracy across all epochs (denoted as the best). The results reported are the means of two independent experiments. Firstly, as evident from the results of the standard CE method in Table 3, the DNN trained solely on CE loss was not able to withstand noisy labels, leading to performance degradation. Secondly, we list the results of certain representative NLL methods with outstanding performance from 2018 to 2022, such as co-teaching, DivideMix, ELR+, UNICON, Mixup, and PENCIL. To thoroughly illustrate the robustness of our method, we specifically compare it with recent NLL methods, including LongReMix, OT-Filter, DISC, ScanMix, C2MT, SLRLNL, RL, PLReMix, and HMW+. Table 3 shows that many SOTA methods achieve excellent and comparable performance under low noise rates, regardless of symmetric or asymmetric noise scenarios (e.g., from 20% to 50% symmetric noise, and 40% asymmetric noise). Nevertheless, our method still achieves optimal performance and significantly outperforms these methods. For instance, in the 20% symmetric/40% asymmetric noise scenario, our method surpasses UNICON, LongReMix, OT-Filter, DISC, ScanMix, C2MT, and PLReMix by margins of 1.0%/1.56%, 0.7%/0.96%, 1.0%/0.51%, 0.9%/1.06%, 1.0%/1.96%, 0.5%/2.7%, and 0.37%/0.55%, respectively. While HMW+ is an improvement based on the UNICON framework, its accuracy did not significantly improve compared with the source framework. In contrast, our method outperforms PLReMix by a noticeable margin. This clearly demonstrates the effectiveness of the two key steps introduced in our approach.

**Figure 6:**
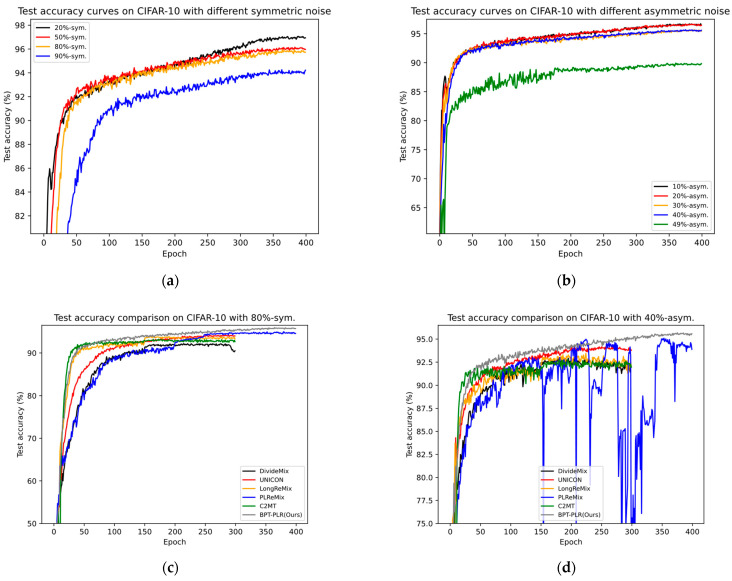
The comparison of test accuracy (%) curves between some SOTA methods and our method using CIFAR-100. (**a**) Test accuracy curves of our method across varying symmetric noise rates. (**b**) Test accuracy curves of our method across varying asymmetric noise rates. (**c**) The comparison of test accuracy between five STOA methods (i.e., DivideMix, UNICON, LongReMix, PLReMix, and C2MT) and our method in the scenario of 80% symmetric noise. These methods were originally set to train for 300 epochs, while our method followed the parameter settings of PLReMix, which are set to 400 epochs. (**d**) The comparison of test accuracy between these STOA methods and our method in the scenario of 30% asymmetric noise.

As the noise ratio increases, the superiority of our method becomes increasingly evident. For example, in scenarios with 90% symmetric/49% asymmetric noise, our method outperforms UNICON, LongReMix, OT-Filter, DISC, ScanMix, and PLReMix by margins of 3.27%/1.9%, 13.2%/5.2%, 3.5%/1.06%, 39%/16.9%, 3%/0.96%, and 2.1%/3.4%, respectively. These methods exhibit varying degrees of overfitting in the 49% asymmetric noise scenario, as indicated by the substantial gaps between the *last* and *best* results, such as 6.6% for LongReMix, 0.9% for OT-Filter, 3.7% for DISC, and 31% for PLReMix. However, our method demonstrates excellent robustness, with a difference of only 0.17%. PLReMix uses flat NCE instead of non-flat NCE to design a flat PLR loss specifically for CIFAR-10 and CIFAR-100, aiming to improve the accuracy of the algorithm, hence referred to as Flat-PLReMix here. However, our approach only employs the original PLR loss across all datasets to demonstrate its robustness. Nevertheless, the performance of our method on CIFAR-10 still significantly surpasses that of Flat-PLReMix. This clearly demonstrates the effectiveness of the two key processes (i.e., BP-GMM and SSO-PLR) proposed in this paper. Furthermore, since many methods only provide results for the 40% asymmetric noise scenario, to thoroughly demonstrate the robustness of our method, we report the results of these methods in the 10% to 30% asymmetric noise scenarios based on publicly available code and compare them with our method. The experimental results further confirm the effectiveness of our method. Finally, in Figure 4, we present the test accuracy curves of our method and some SOTA methods. It can be observed from the figures that our method maintains steady progress, demonstrating its effectiveness in resisting noisy labels as training progresses. Combining Figure 6 with Figure 3 and Figure 5, it is evident that our proposed BP-GMM process divides the data into labeled subsets, with sizes closer to the true clean rate. Within the labeled subset, there are more TP samples for each category, while the number of FP samples is relatively low. As a result, we achieve better test performances. Since the SSO-PLR technique proposed in this paper combines oversampling strategies with PLR loss to extract more information from unlabeled samples, it enhances multiple learning on clean samples, accelerating convergence speed (i.e., the steeper test accuracy curves in Figure 6c,d) and enhancing final test results.

#### 4.2.2. Results on CIFAR-100

To further highlight the advantages of our method, we validate our approach on the CIFAR-100 dataset with varying synthetic noisy labels, which is the same as previous SOTA methods. Table 4 provides a comparison of our method with some SOTA methods on this dataset. Consistent with the experiments on CIFAR-10, we also present both the “last” and “best” results. Firstly, from the testing results of CE, co-teaching, Mixup, and PENCIL in scenarios where the noise ratio is greater than or equal to 30%, regardless of symmetric or asymmetric noise, it can be observed that as the number of classes increases, the impact of noisy labels on the model becomes more severe. DNNs trained with these methods almost lose discriminative ability and are replaced by random guessing (i.e., the test accuracies of these methods are below 50%). Furthermore, recent SOTA methods such as DivideMix, UNICON, DISC, LongReMix, RL, C2MT, PLReMix, and HMW+ have achieved significant improvements on CIFAR-100 across varying noise scenarios. However, in most scenarios, our method still shows improvements over these methods. For instance, in low-noise-rate scenarios, specifically 20%-sym. And 20%-asym., our method leads by 1.55%/9.5%, 0%/0.84%, 0.1%/0.64%, 1.85%/-, 0.06%/−0.68%, 1.35%/1.2%, 0.9%/-, and 2.2%/2.4%, respectively. Notably, RL utilizes a deeper ResNet-34 for training on this set, while the difference in the results between PreAct ResNet-18 and ResNet-34 for PENCIL shows that as the model gets deeper, the test performance improves. Nevertheless, our method still achieves comparable or slightly superior results to RL, indicating its advantage. Similar to observations on CIFAR-10, as the noise ratio increases, our method continues to maintain optimal (e.g., in 50%-sym., 80%-sym., 10%, and 30%-asym. Scenarios) or near-optimal results (e.g., second only to ScanMix in the 90%-sym. Scenario), except for the 40%-asym. Scenario. In this case, our method lags behind RL, OT-Filter, DISC, etc., by nearly 2.2%.

**Figure 7:**
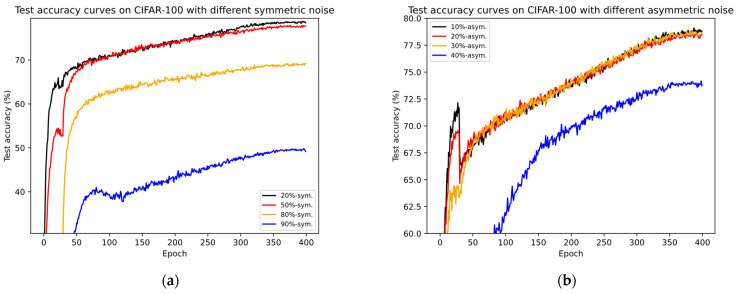

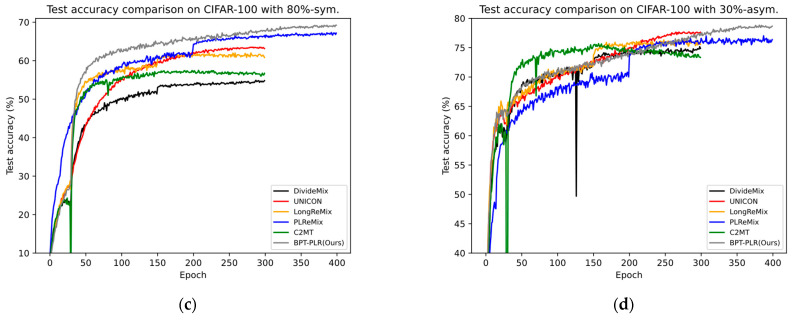
The comparison of test accuracy (%) curves between some SOTA methods and our method using CIFAR-100. (**a**) Test accuracy curves of our method across varying symmetric noise rates. (**b**) Test accuracy curves of our method across varying asymmetric noise rates. (**c**) The comparison of test accuracy between five STOA methods (i.e., DivideMix, UNICON, LongReMix, PLReMix, and C2MT) and our method in the scenario of 80% symmetric noise. These methods were originally set to train for 300 epochs, while our method followed the parameter settings of PLReMix, which are set to 400 epochs. (**d**) The comparison of test accuracy between these SOTA methods and our method in the scenario of 30% asymmetric noise.

Our analysis suggests that the main issue lies in these methods adjusting hyperparameters dynamically based on noise types and ratios, while we maintain nearly consistent parameter settings across all experiments. Additionally, as the asymmetric noise ratio on CIFAR-100 rises to 40%, the number of clean and noisy samples per class becomes almost equal (300:200), posing a challenge to calculating a reliable negative set for PLR loss. It is one of our focal points for future research. Despite this, our method still achieves suboptimal performance compared with UNICON, far outperforming LongReMix, SLRLNL, HMW+, and others. Moreover, such extreme cases are rare in real-world scenarios, as most datasets have a large number of samples per class (greater than 1000), resulting in a significant gap between the numbers of clean and noisy samples per class, even with large categories and high noise ratios. Therefore, we can conclude that our method is suitable for most noisy scenarios with a large number of categories and demonstrates good robustness and classification performance. Similarly, in Figure 7, we present the test accuracy curves of our method and some SOTA methods. It can be observed from the figures that our method maintains steady progress, demonstrating its effectiveness in resisting noisy labels as training progresses. Combining the results of Figure 5c,d, it can be seen that the sizes of the unlabeled subsets we partitioned (referred to as noisy label sets) are closer to the true noise rates. The analysis combining Figure 3 and Figure 5 clearly shows that both the BP-GMM process and the SSO-PLR process still have a certain effect on noisy datasets with a larger number of categories. Therefore, the test curve of our method is steeper and higher compared with the test accuracy curves of several SOTA methods shown in Figure 7c,d.

### 4.3. Experiments on Real-World Noisy Datasets

We have conducted extensive experiments on the CIFAR-10 and CIFAR-100 datasets, demonstrating the effectiveness of our method. In this section, we apply it to two real-world noise datasets crawled from websites to further validate its performance. We conducted experiments on the Animal-10N and Clothing1M datasets, and the experimental analysis is below.

#### 4.3.1. Results on Animal-10N

Since SOTA methods mainly employ two network architectures (e.g., 9-layer CNN and VGG-19N) for Animal-10N evaluation, we simultaneously provided the test results of the BPT-PLR method based on these two networks in Table 5. Additionally, we reported the results of LongReMix and PLReMix using publicly available code on this dataset. Due to PLReMix utilizing the original PLR loss on real-world noisy datasets in the reference, it is denoted as N-Flat-PLReMix (non-flat PLReMix). From the table, it is evident that our method achieved the best performance across two network architectures. Our method outperforms TCC-net and C2MT by 4.0% and 2.5%, respectively, on the 9-layer CNN, and surpasses OT-Filter, DISC, LongReMix, C2MT, SLRLNL, and HMW+ by at least 1% on Vgg-19N. Although the best accuracy of PLReMix is close to ours (with a difference of approximately 0.3%), its last accuracy significantly lagged behind our method (with a difference of approximately 0.75%).

**Figure 8:**
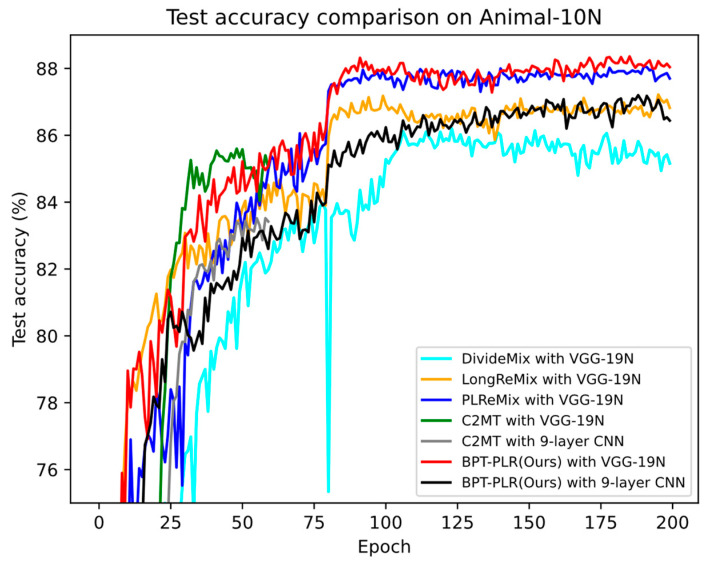
The comparison of test accuracy (%) curves between reproduced methods and our method on Animal-10N.

Through the comparison experiments on Animal-10N, we further illustrated the two advantages of our method: maintaining stable and excellent performance across various noise scenarios and being insensitive to model structures, thus being compatible with most DNN networks. In Figure 8, we present the test accuracy curves of our method and some reproduced methods on this dataset. Similar to Figure 6 and Figure 7, we still find that the test accuracy curve of our proposed method is steeper and higher than existing SOTA methods, which fully demonstrates its effectiveness in dealing with real-world fine-grained noisy datasets.

#### 4.3.2. Results on Clothing1M

Table 6 presents the experimental results on the Clothing1M dataset. From the table, it can be observed that our method performs slightly worse than existing state-of-the-art methods. The core issue lies in adopting almost identical hyperparameter settings as those used on the Animal-10N dataset. Additionally, due to our adherence to the PLReMix approach, we randomly sample 64K data points for training at each epoch. Consequently, the BP-GMM process faces potentially different training sets in each epoch, diminishing the coherence of balanced partitioning. This inconsistency affects both the partition accuracy and subsequent PLR loss computation, resulting in a slight decrease in performance. Furthermore, while PLReMix slightly outperforms our approach, this advantage stems from its selective use of flat PLR and non-flat PLR tailored to different datasets. In contrast, we employed the same non-flat PLR loss across all datasets. Despite this, we still achieved near-excellent performance, trailing the SOTA method (i.e., PLReMix, OT-Filter, and C2MT) by only approximately 0.1–0.2%. Considering that the Clothing1M dataset contains 1 million training samples, this performance gap can be considered negligible. Furthermore, we still outperformed many recent methods such as UNICON, DISC, and SLRLNL. Therefore, our proposed method is applicable to large-scale noisy datasets.

### 4.4. Ablation Study

In this section, we conduct an ablation analysis on several key modules proposed in this paper to fully demonstrate their efficacy. Compared with the original PLReMix method, this paper mainly introduces two key processes: BP-GMM and SSO-PLR. In BP-GMM, we combine balanced partitioning with a two-dimensional GMM and perform sample selection based on both label and semantic information. Therefore, in the ablation experiments, we regard the balanced partitioning module as a key module, abbreviated as BP. Similarly, in SSO-PLR, we treat oversampling techniques and PLR loss as two key modules, abbreviated as OS and PLR, respectively. We present the ablation experiment results on several key modules in Table 7. If the BP and OS columns are marked as “✗” in the corresponding experiment result row, it indicates that the corresponding module was not used in that experiment, and vice versa. The PLR column is slightly different; if marked as “✗”, it indicates that we used the original CRL loss for both labeled and unlabeled samples, meaning the reliable negative class set $O_{i}^{t}$ (Equation (14)) was not constructed; otherwise, it indicates the use of non-flat PLR loss (just utilized in all datasets, unlike PLReMix, where flat and non-flat PLR losses are dynamically employed based on dataset types). Analyzing the results in Table 7, we draw the following conclusions:

The effect of each module. From Rows #1 to #4 in Table 7, it is evident that using each module individually (such as Row #2 for BP, Row #3 for OS, and Row #4 for PLR) improved the average testing accuracy compared with the original method (i.e., Row #1) and also increased the risk of model overfitting. For instance, in Rows #3 (80%-sym.) and #4 (40%-asym.), the *last* results significantly lag behind the *best*, indicating the overfitting of DNNs in the later stages of training. This suggests that while individual modules enhance the model’s robustness, their stability still needs improvement.

The effect of combining BP and OS. Although using OS alone may lead to model overfitting, we have demonstrated that combining it with BP results in mutual influence between the two modules, significantly enhancing the model’s robustness and consistently improving testing accuracy. By comparing Rows #1 and #5, we observed that in two distinct noise scenarios, the combination of BP and OS improved performance by 0.9%/0.89% and 6.4%/6.3%, respectively. Additionally, the average testing accuracy increased by 3.2%/3.6%. This clearly underscores the necessity of utilizing both BP and OS modules simultaneously. Subsequently, comparing the results of using both BP and OS (Row #5) with those of using BP or OS alone (Rows #2 or #3), we found that introducing OS benefits the BP operation, further enhancing the performance of the model.

The effect of combining BP and PLR**.** Similar to the performance of OS, using PLR alone can lead to overfitting in scenarios with noisy labels. However, experiments in Row #6 demonstrated that combining PLR with BP can overcome this issue and consistently enhance the model’s robustness. Comparing the results of Rows #1 and #6, it is evident that in two different noise scenarios, this combination improves performance by approximately 0.1%/0.1% and 7%/7% compared with the original method. Additionally, the average testing accuracy Is Increased by 3.2%/3.6%. Furthermore, by comparing the results of Row #5 with Rows #2 or #4, we further confirm the necessity of combining BP and PLR.

The effect of combining OS and PLR. Similar to the experimental analysis above, when OS and PLR are combined, the testing performance of DNNs is significantly improved compared with the original method. Comparing the results of Rows #1 and #7, the combination improves performance by approximately 0.8%/0.7% and 2.3%/2.2%, respectively. Compared with Rows #3 and #4, although the improvement in testing performance of the two combinations is negligible, they mitigate the overfitting issues caused by using these two components separately, demonstrating the necessity of using OS and PLR simultaneously.

The effect of combining BP, OS, and PLR. Finally, we compare the results of using all three components introduced in this paper (i.e., the BPT-PLR framework, Row #8) with the optimal results from several other ablation experiments (i.e., Row #5). It was found that our method successfully overcame various issues mentioned above. It not only applies to scenarios with both asymmetric and symmetric noise but also enables the model to consistently maintain robustness and achieve optimal performance. Although our method performed slightly worse by 0.1% compared with using only BP and OS in the symmetric noise scenario, it outperformed other models by approximately 0.8% in the asymmetric noise scenario, resulting in a better average outcome than that of the experimental method. This fully demonstrates the necessity of using all three components simultaneously.

**Figure 9:**
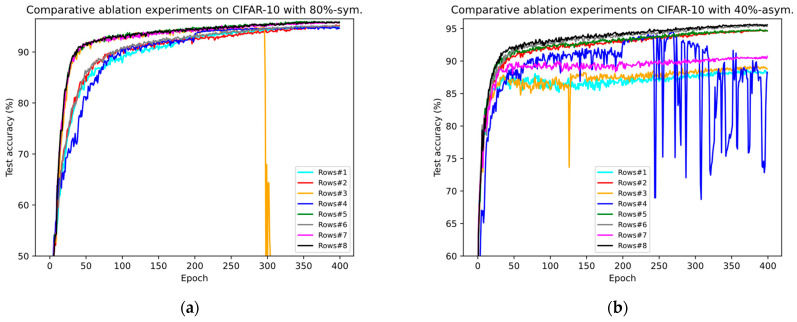
Comparative ablation experiments for our method on CIFAR-10 with synthetic noisy labels. (**a**) Test accuracy (%) comparisons of different module combinations on CIFAR-10 with 80% symmetric noise. “Rows#i” of the figure refers to the *i*-th row in Table 7. (**b**) Test accuracy (%) comparisons of different module combinations on CIFAR-10 with 40% asymmetric noise.

These experiments have analyzed the impact of each component introduced in this paper on the model’s testing performance in different noise scenarios. Through quantitative analysis, we found that the more components introduced, the more stable the model’s robustness. When all components are used simultaneously, we can obtain nearly optimal results, demonstrating the necessity of the framework proposed in this paper. Furthermore, Figure 9 presents the results of each ablation experiment in the form of testing accuracy curves, providing a more visual comparison of the changes in accuracy. Clearly, the BPT-PLR framework proposed in this paper (i.e., Row #8) maintains stable performance and achieves the best testing accuracies. Furthermore, it is evident that Rows #3 (using only OS) and #4 (using only PLR) in Figure 9a,b, during the late stages of training, begin to overfit on samples with noisy labels, resulting in a dramatic decline in test performance. This also explains the significant difference between the “*last*” and “*best*” results corresponding to these two methods in Table 7. This further illustrates the necessity of simultaneously utilizing the three modules proposed in this paper for the BPT-PLR method.

## 5. Discussion

We validated the effectiveness of our proposed method through extensive experiments on four benchmark datasets. The comparative experiments shown in Table 3 and Table 4 demonstrate the superior performance of our method on synthetic noise datasets, indicating its applicability to both fine-grained and coarse-grained noisy datasets. We illustrate the necessity of the proposed BP-GMM process in Figure 3, showing that it can improve the balance of labeled subsets after partitioning, increase the number of TP samples, and maintain or even reduce the FP samples. Additionally, we elaborated on the necessity of oversampling techniques in the SSL-based sample selection framework, as shown in Figure 5. Finally, in Table 5 and Table 6, we provide the results of our approach on two real-world noisy datasets and compare them with several SOTA methods, further demonstrating the effectiveness of our framework. Moreover, we verified the robustness of our method to network structures and demonstrated its applicability to most DNN models, showcasing its broad utility. In Figure 6, Figure 7 and Figure 8, we compare the test accuracy curves of our method with those of several SOTA methods, revealing not only a faster convergence rate (steeper curve) but also higher test performance. Combining the contents of Figure 3 and Figure 5 further emphasized the necessity of the proposed two key processes. Finally, through extensive ablation experiments, we affirmed the effectiveness of several core modules utilized in the proposed key processes. From the ablation experiments, it is evident that although individual modules may not consistently improve the model’s test performance, when used together, they mutually enhance and stabilize the model’s test performance, underscoring the indispensability of these key modules.

Naturally, BPT-PLR has some limitations. For instance, as shown in Table 4, our method does not outperform existing methods in handling 40% asymmetric noise and 90% symmetric noise. Although such extreme noise scenarios are uncommon in practice, we still consider them as a focus for future research. Additionally, while we significantly outperformed existing methods on Animal-10N, we only marginally matched SOTA methods on Clothing1M, failing to surpass them completely. This indicates that while our method demonstrates certain robustness against various noise datasets, the robustness level is not consistently stable, which is also a point of consideration for future work. Finally, we plan to extend the two key processes proposed in this paper to the Out-of-Distribution (OOD) sample detection domain.

## 6. Conclusions

As over-parameterized deep neural networks (DNNs) attempt to fit all samples, including noisy labels, they tend to overfit, which compromises their generalization ability. In this paper, we propose a balanced partitioning and training framework with pseudo-label relaxed contrastive loss (BPT-PLR) to address the challenge of noisy label learning. It aims to reduce the impact of noisy labels on DNNs and improve classification performance. BPT-PLR leverages two crucial processes: balanced partitioning with a two-dimensional Gaussian Mixture Model (BP-GMM) and semi-supervised oversampling training with pseudo-label relaxed contrastive loss (SSO-PLR). BP-GMM identifies noisy labels based on semantic and class information, while SSO-PLR combines PLR with SSL techniques to improve model robustness and avoid conflicts with supervised losses. We validate the effectiveness of BPT-PLR on four benchmark datasets in the NLL domain, demonstrating its optimal or near-optimal performance compared with SOTA methods. We hope this work will inspire further research on sample selection methods for NLL via these two key processes.

## Figures and Tables

**Table 1:** Overview of the datasets.

Name	Class Number	Training Number	Testing Number	Original Size	Cropped Size
CIFAR-10	10	50*K*	10*K*	32 × 32	32 × 32
CIFAR-100	100	50*K*	10*K*	32 × 32	32 × 32
Animal-10N	10	50*K*	5*K*	64 × 64	64 × 64
Clothing1M	14	1*M*	10*K*	256 × 256	224 × 224

**Table 2:** The experimental settings of our method.

Dataset	CIFAR-10	CIFAR-100	Clothing1M	Animal-10N
Backbone	PreAct ResNet-18		ResNet-50	VGG19-BN/9-layer CNN
*lr*	0.02	0.02	0.01	0.01
Optimizer	SGD	SGD	SGD	SGD
Weight decay	5 × 10^−4^	5 × 10^−4^	1 × 10^−3^	1 × 10^−3^
Momentum	0.9	0.9	0.9	0.9
b	64	64	64	128
$E_{w}$	10	30	5	30
$E_{tot}$	400	400	80	200
β	4	4	0.5	4

**Table 3:** The comparison of test accuracies (%) using CIFAR-10 across various noisy scenarios. The best accuracies are shown in bold. Underlines indicate reproduced results. “†“ denotes that the backbone is ResNet-32.

Methods		The Comparison of Test Accuracies (%) on CIFAR-10								
		Symmetric Noise				Asymmetric Noise				
		20%	50%	80%	90%	10%	20%	30%	40%	49%
Standard CE		86.8	79.4	62.9	42.7	88.8	86.1	81.7	76.1	-
Co-teaching [25] (18)		86.5	76.1	25.4	-	87.2	-	84.7	75.7	-
Mixup [16] (18)		95.6	87.1	71.6	52.2	93.3	88.0	83.3	77.7	-
PENCIL [20] (19)		92.4	89.1	77.5	58.2	93.1	92.9	92.6	91.6	-
DivideMix [6] (20)	last	95.7	94.4	92.9	75.4	-	-	-	92.1	76.3
	best	96.1	94.6	93.2	76.0	-	-	-	93.4	84.7
ELR+ [27] (20)		95.8	94.8	93.3	78.7	95.4	94.7	94.7	93.0	-
UNICON [28] (22)		96.0	95.6	93.9	90.8	95.3	-	94.8	94.1	87.1
LongReMix [8] (23)	last	96.0	94.8	93.3	79.1	95.4	94.1	93.5	94.3	77.8
	best	96.3	95.1	93.8	79.9	95.6	94.6	94.3	94.7	84.4
OT-Filter [30] (23)	last	-	-	-	-	95.2	94.9	94.5	-	87.7
	best	96.0	95.3	94.0	90.5	95.6	95.2	94.9	95.1	88.6
DISC [31] (23)	last	-	-	-	32.3	96.2	95.7	95.2	-	69.0
	best	96.1	95.1	84.7	55.8	96.3	95.8	95.3	94.6	72.7
ScanMix [39] (23)		95.7	93.9	92.6/93.5	90.3	-	-	-	93.4	87.1
RL † [24] (23)	last	-	90.57	-	61.72	93.80	93.51	93.05	92.31	-
	best	-	90.73	-	62.32	94.21	93.86	93.23	93.57	-
TPCR † [32] (24)		93.2	-	86.9	-	-	93.3	92.3	91.0	-
Flat-PLReMix [7] (24)	last	96.46	95.36	94.84	91.54	-	-	-	94.72	55.1
	best	96.63	95.71	95.08	91.93	-	-	-	95.11	86.2
C2MT [9] (24)	last	96.1	94.8	92.8	-	95.1	93.0	93.5	92.6	-
	best	96.5	95.0	93.4	-	95.4	94.3	94.1	92.9	-
SLRLNL [33] (24)		92.5	-	78.9	-	-	93.1	92.5	92.0	-
HMW+ [29] (24)		93.5	95.2	93.7	90.7	93.5	-	94.7	93.7	-
BPT-PLR (Ours)	last	**96.89**	**96.03**	**95.45**	**93.84**	**96.61**	**96.49**	**95.63**	**95.51**	**89.49**
	best	**97.00**	**96.16**	**95.66**	**94.07**	**96.76**	**96.68**	**95.82**	**95.66**	**89.66**

**Table 4:** The comparison of test accuracies (%) on CIFAR-100 across various noisy scenarios. The best accuracies are shown in bold. Underlines indicate reproduced results. “†“ denotes that the backbone is ResNet-34. “x/x” means the last/best accuracies.

Methods		Test Accuracy (%) on CIFAR-100							
		Symmetric Noise				Asymmetric Noise			
		20%	50%	80%	90%	10%	20%	30%	40%
Standard CE		62.0	46.7	19.9	10.1	68.1	63.6	53.5	44.5
Co-teaching [25] (18)		49.2	35.1	5.7	-	54.1	-	49.6	43.7
Mixup [16] (18)		67.8	57.3	30.8	14.6	72.4	65.1	57.6	48.1
PENCIL [20] (19)		69.4	57.5	31.1	15.3	76.1	68.9	59.3	48.3
PENCIL † [20] (19)		73.86	-	-	-	75.93	74.70	72.52	63.61
DivideMix [6] (20)		76.9/77.3	74.2/74.6	59.6/60.2	31.0/31.5	69.5	69.2	68.3	51.0
ELR+ [27] (20)		77.6	73.6	60.8	33.4	77.4	75.5	75.1	74.0
UNICON [28] (22)		78.9	77.6	63.9	44.8	78.2	-	75.6	74.8
OT-Filter [30] (23)		76.7	74.6	61.8	42.8	-	-	-	**76.5**
DISC [31] (23)		78.8	75.2	57.6	-	78.1/78.4	77.5/77.2	76.3/76.8	**76.5**
LongReMix [8] (23)		77.5/77.9	74.9/75.5	61.7/62.3	30.7/34.7	-	-	-	54.9/59.8
ScanMix [39] (23)		76.0/77.0	75.4/75.7	65.0/66.0	**58.2/58.5**	-	-	-	-
RL † [24] (23)		78.79	-	49.81	-	**79.72**	**79.20**	**79.04**	**76.50**
TPCR † [32] (24)		74.8	-	53.1	-	-	77.2	75.4	71.3
C2MT [9] (24)		76.5/77.5	73.1/74.2	57.5/57.7	-	77.1/77.8	77.3/77.7	74.5/75.7	-
SLRLNL [33] (24)		69.4	-	32.6	-	-	72.5	71.9	69.7
Flat-PLReMix [7] (24)		77.78/77.95	77.31/77.78	68.76/68.41	49.44/**50.17**	-	-	-	-
HMW+ [29] (24)		76.6	75.8	63.4	43.4	76.6	-	76.3	72.1
BPT-PLR (Ours)	*last*	**78.66**	**77.77**	**69.06**	**49.49**	**78.68**	**78.30**	**78.52**	73.95
	*best*	**78.85**	**78.02**	**69.31**	**49.85**	**79.04**	**78.54**	**78.82**	74.30

**Table 5:** A comparison of test accuracies (%) on Animal-10N. The best accuracies are shown in bold. Underlines indicate reproduced results. “x/x” means the last/best accuracies. “†“ denotes that the backbone is ResNet-34.

Methods		Test Accuracy (%)
Training with 9-layer CNN		
Standard		82.68
Co-teaching [25] (18)		82.43
JoCoR [46] (20)		82.82
TCC-net [47] (23)		83.22
C2MT [9] (24)		84.30/84.76
Ours	last	**86.79**
	best	**87.20**
Training with Vgg-19N		
Mixup [16] (18)		82.7
SELFIE [44] (19)		81.8
DivideMix [6] (20)		85.35/86.20
OT-Filter [30] (23)		85.5
DISC [31] (23)		87.1
LongReMix [8] (23)		86.88/87.22
TPCR † [32] (24)		87.39
C2MT [9] (24)		85.8/85.9
SLRLNL [33] (24)		86.4
N-Flat-PLReMix [7] (24)		87.27/88.0
HMW+ [29] (24)		86.5
BPT-PLR (Ours)	last	**88.02**
	best	**88.28**

**Table 6:** The comparison of test accuracies (%) on Clothing1M. “*” indicates the backbone is PreAct ResNet-18. Underlines indicate reproduced results. The top-3 results are shown in bold.

Methods	Test Accuracy (%)
Standard	68.94
Co-teaching * [25] (18)	69.21
CJC-net * [26] (21)	72.71
TCC-Net * [47] (23)	70.46
Co-teaching [25] (18)	71.70
PENCIL [20] (19)	73.49
Divide-Mix [6] (20)	74.21
ELR+ [27] (20)	74.39
ECMB [2] (21)	73.29
UNICON [28] (22)	74.00
LongReMix [8] (23)	74.38
ScanMix [39] (23)	74.35
DISC [31] (23)	73.72
OT-Filter [30] (23)	**74.50**
RL [24] (23)	74.29
C2MT [9] (24)	**74.45**
PLM [48] (24)	73.30
Ultra+ [49] (24)	74.03
N-Flat-PLReMix [7] (24)	**74.58**
SLRLNL [33] (24)	74.15
BPT-PLR (Ours)	74.37

**Table 7:** Ablation studies of our method. The best accuracies are shown in bold and we report **last/best** results where “✗” indicates the module is not employed while “✓“ indicates the opposite. “BP” represents a balanced partitioning module, and “OS” represents an oversampling module. The column “PLR” indicates the usage of CRL loss if it is “✗”; otherwise, the PLR loss described in this paper is employed. “✗” represents the mean results between 80%-sym. And 40%-asym. Row #4 indicates the original PLReMix. Each result comes from one experiment.

		Noise Types		Last/Best Test Accuracy (%)		
Modules				CIFAR-10		
Rows	BP	OS	PLR	80%-sym.	40%-asym.	Average Accuracy
1	✗	✗	✗	94.94/95.10	88.28/88.61	91.61/91.86
2	✓	✗	✗	95.14/95.31	94.67/94.78	94.91/95.04
3	✗	✓	✗	10.00/94.82	88.99/89.19	50.00/92.01
4	✗	✗	✓	94.72/94.98	79.94/94.55	87.33/94.77
5	✓	✓	✗	**95.83/95.99**	94.68/94.90	95.26/95.45
6	✓	✗	✓	95.06/95.18	95.37/95.54	95.22/95.36
7	✗	✓	✓	**95.78/95.88**	90.58/90.85	93.18/93.37
8	✓	✓	✓	**95.77/95.95**	**95.51/95.69**	**95.64/95.82**

## Data Availability

The datasets, such as CIFAR-10/100, Clothing1M, and Animal-10N, employed in the current study are available at http://www.cs.toronto.edu/~kriz/cifar.html (accessed on 1 April 2024), https://github.com/Cysu/noisy_label (accessed on 1 April 2024), https://dm.kaist.ac.kr/datasets/animal-10n/ (accessed on 1 April 2024), respectively.
